# Psychological Resilience and Frailty Progression in Older Adults

**DOI:** 10.1001/jamanetworkopen.2024.47605

**Published:** 2024-11-25

**Authors:** Bo Ye, Yunxia Li, Zhijun Bao, Junling Gao

**Affiliations:** 1 Shanghai Key Laboratory of Clinical Geriatric Medicine, Huadong Hospital Affiliated to Fudan University, Shanghai, China; 2 Department of Preventive Health Care, Heqing Community Health Service Center, Shanghai, China; 3 School of Public Health, Fudan University, Shanghai, China

## Abstract

**Importance:**

The association between psychological resilience (PR) and frailty progression in older adults is not fully understood.

**Objective:**

To investigate the longitudinal associations between PR and its changes (ΔPR) with frailty progression among older adults.

**Design, Setting, and Participants:**

Data were collected from a community-based prospective cohort study conducted in Shanghai from 2020 to 2023. Older adults aged 60 years and above were recruited at baseline and completed follow-up assessments. Statistical analysis was performed from May to July 2024.

**Exposures:**

PR was assessed using the 25-item Connor-Davidson resilience scale (CD-RISC). ΔPR was calculated using the difference between 3-year follow-up and baseline CD-RISC scores. Multiple key covariates were included such as demographics, health behaviors, social participation, and chronic diseases.

**Main Outcomes and Measures:**

Frailty was evaluated using the Chinese Frailty Screening Scale-10. Frailty progression was categorized into 3 groups (improvement, stability, and deterioration) by the difference assessment between 3-year follow-up and baseline.

**Results:**

A total of 4033 participants were included in the study (mean [SD] age, 71.0 [6.1] years; 2257 [56.0%] women). Over a 3-year follow-up period, 1142 participants (28.3%) experienced an improvement in frailty, 1200 (29.8%) experienced stability, and 1691 (41.9%) exhibited deterioration. After controlling for confounding variables, a significant association was found between PR and future frailty, particularly when baseline frailty scores were at least 3 vs 2 or lower (β, −0.136 [95% CI, −0.214 to −0.057] vs −0.020 [-95% CI, −0.052 to 0.012]; *P* for interaction < .001). A similar association was observed between PR and frailty improvement (adjusted odds ratio [OR], 1.28 [95% CI, 1.05 to 1.57]). Furthermore, ΔPR displayed a significant association with frailty, exhibiting a nonlinear association that was stronger when ΔPR was less than 0 vs when ΔPR was greater than 0 (β,−0.186 [95% CI, −0.228 to −0.145] vs −0.068 [95% CI, −0.107 to −0.029]; *P* for nonlinear < .001). Similarly, ΔPR was significantly associated with frailty progression, indicating both improvement (adjusted OR, 1.30 [95% CI, 1.19 to 1.42]) and deterioration (adjusted OR, 0.74 [95% CI, 0.69 to 0.80]). These associations were more pronounced among older adults with higher levels of baseline frailty.

**Conclusions and relevance:**

In this cohort study of community-dwelling older adults, a longitudinal association between PR and frailty progression was found. The results suggest that monitoring changes in PR can help forecast future frailty trajectories, particularly highlighting the need to support individuals facing declines in resilience. Targeted interventions that prioritize enhancing PR have potential to prevent and ameliorate frailty.

## Key Points

**Question:**

Is psychological resilience (PR) associated with the development of frailty among community-dwelling older adults?

**Findings:**

In this cohort study involving 4033 participants over a 3-year period, higher levels of PR were associated with a reduction in frailty, particularly among those with initially higher frailty levels. Furthermore, changes in PR were associated with the progression of frailty, demonstrating a nonlinear association.

**Meaning:**

These results suggest that PR may represent a valuable target for interventions, as enhancing resilience holds potential for preventing and reserving frailty among older adults.

## Teaser

This cohort study assesses longitudinal associations between psychological resilience and its changes with frailty progression among older adults in Shanghai, China.

## Introduction

With the rapid and substantial aging of the population in China,^1^ there is a pressing need to identify factors that contribute to healthy aging. Researchers have concentrated on pinpointing markers of aging and related deficits to guide the development of clinical interventions and the allocation of resources.^2^ Frailty represents the most challenging aspect of population aging. It is characterized by a state of increased risk for ineffective homeostasis following a stressor event, resulting from the cumulative decline of various physiological systems over a person’s lifetime.^3^ Individuals exhibiting frailty face an increased risk of adverse outcomes, such as falls, hospitalizations, disability, and mortality.^4^

Recent research has aimed to identify factors that promote healthy aging^5^ and resilience,^6,7^ which is understood as an individual’s ability to bounce back after experiencing adversity.^8^ In the context of aging, individual differences in the aging process can be conceptualized as an accumulation of wear and tear from daily experiences and major life stressors, interacting with genetic predispositions and early life experiences.^9^ Both physical and psychological resilience are crucial metrics for coping with the challenges of aging. Physical resilience is characterized by the maintenance or recovery of physical function following biomedical or physiological challenges,^10^ whereas psychological resilience (PR) is defined as the ability to recover swiftly from difficulties.^11^

Theoretically, resilient older adults can readily return to a baseline state, demonstrating the ability to bounce back and how trauma exposed individuals failed to exhibit negative consequences, which indicates their resistance to adversity. Previous studies have identified associations between PR and enhanced physical function, as well as better outcomes following hip fractures, surgical procedures, and intensive care unit admissions.^12,13,14^ More broadly, research involving community-dwelling older adults has shown that greater PR was correlated with more favorable health transitions and trajectories, including self-rated health, functional limitations, disability, and depressive symptoms.^15^ A secondary analysis of a multicenter randomized clinical trial (RCT) also found that greater PR was associated with a lower body mass index, fewer hospitalizations, faster gait speed, greater grip strength, and lower likelihood of frailty among older adults with type 2 diabetes.^16^ Additionally, several cross-sectional studies have indicated a significant association between PR and frailty across various patients and settings.^17,18,19,20,21,22,23^ While Costenoble et al^24^ found that the correlations between PR and its changes with frailty transitions after 1 year were not significant in a sample of 322 octogenarians, the inconsistent result may be limited by the short observational period and the small simple size.^24^ Moreover, a review has indicated that lifestyle and obesity were related to frailty transitions, while limited studies examined their psychological and social factors.^25^ In summary, there remains a substantial gap in our understanding of the influence of PR on frailty progression.

In this study, we aimed to find a longitudinal association between PR and frailty using a 3-year, community-based prospective cohort with a large sample size. Furthermore, given the dynamic nature of frailty and the 2 core attributes of PR, we hypothesized that PR and its changes would be associated with frailty progression, and that these associations would vary based on different initial levels of frailty.

## Methods

### Study Design and Participants

**Figure 1.**
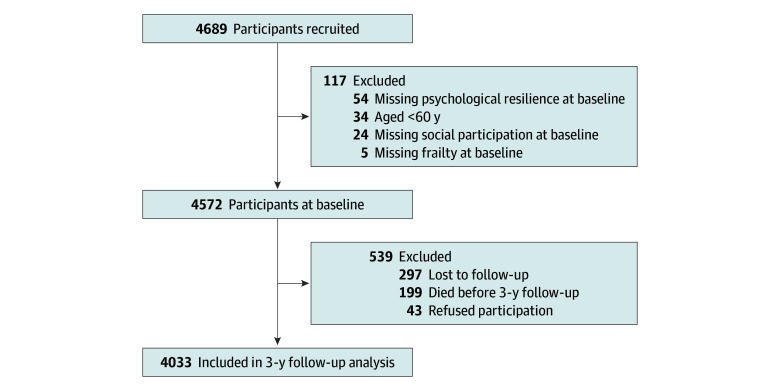
Flowchart of Study Participants

This community-based prospective cohort study was conducted among older adults in Shanghai, China. The baseline survey (wave 1) took place between June and December 2020, followed by the first (wave 2) and second (wave 3) follow-ups conducted in the latter parts of 2021 and 2023, respectively. Participants aged 60 years and above were randomly selected using a 2-stage sampling method. Sixteen communities were chosen for convenience, resulting in the recruitment of 4689 older adults. To qualify for participation, individuals had to be 60 years or older, free of progressive tumors or severe mental disorders, and without any expression or communication disorders. Trained interviewers visited participants in their homes or invited them to community health care centers to conduct face-to-face surveys with a self-administered electronic questionnaire. Out of 4572 older adults who completed the full survey at baseline, 4033 individuals with complete data at baseline and after 3 years were included in this analysis (Figure 1). The human participants’ research protocol received ethical review and approval from the Research Ethics Committee of the Medical Research at the School of Public Health, Fudan University. Prior to enrollment, written informed consent was obtained from either the participants themselves or from family members for illiterate older individuals. This report adhered to the Strengthening the Reporting of Observational Studies in Epidemiology (STROBE) reporting guideline for cohort studies.

### Frailty Assessment

Frailty was assessed using the Chinese Frailty Screening Scale-10 (CFSS-10),^26^ which consists of 10 items that evaluate both physical components of frailty—such as exhaustion, resistance, and physical inactivity—and cognitive aspects, including attention, orientation, and depressive symptom (eTable 1 in Supplement 1). Each question is scored as 0 (no) or 1 (yes), resulting in total scores ranging from 0 to 10, where higher scores indicate greater severity of frailty. The CFSS-10 was developed and validated among Chinese older adults, with consideration also given to the framework of healthy aging.^26^ In this study, the CFSS-10 exhibited acceptable internal consistency, with Cronbach α values of 0.582 at baseline and 0.766 at follow-up.

### Psychological Resilience Assessment

PR was evaluated using the Connor-Davidson Resilience Scale (CD-RISC),^11^ a self-report questionnaire consisting of 25 statements such as “Able to adapt to change,” “Can deal with whatever comes,” and “Coping with stress strengthens” (eTable 2 in Supplement 1). Participants rated their responses on a 5-point Likert scale, ranging from 0 (not true at all) to 4 (true nearly all of the time). Total scores varied between 0 and 100, with higher scores reflecting a greater degree of resilience. The CD-RISC is widely recognized for measuring resilience and exhibiting robust psychometric properties, making it suitable for use in older adults.^27^ It has also been translated into Chinese, showing excellent reliability and validity.^28^ In this study, the CD-RISC demonstrated excellent internal consistency, with Cronbach α values of 0.968 at baseline and 0.979 at follow-up.

### Covariates

Participants’ basic information included age, gender, marital status, education, monthly income, smoking status, and alcohol consumption. Physical exercise was evaluated using 2 questions: older adults who reported doing moderate physical activities 3 days or more per week for a duration of 30 minutes or longer were classified as exercise regularly, whereas those reporting 0 days of activities were classified as having no exercise, and the rest were categorized as irregular exercisers. Detailed classification is shown in Table 1. Social participation was assessed using an 8-item questionnaire,^29^ yielding a total score ranging from 0 to 32, with a Cronbach α of 0.760 in this study. Additionally, participants self-reported the presence of up to 20 chronic diseases, including hypertension, diabetes, coronary heart disease, stroke, arrhythmia, myocardial infarction, congestive heart failure, cervical spondylosis, herniated disc, arthritis, osteoporosis, asthma, chronic lung disease, chronic kidney disease, digestive diseases, thyroid disease, Parkinson disease, cancer, dementia, and epilepsy.

**Table 1.** Participant Characteristics and Comparison of Frailty Progression by Subgroups

Variables	Participants, No. (%)	Frailty progression, No. (%)			*P* value
		Improvement	Stability	Deterioration	
Overall	4033 (100.0)	1142 (28.3)	1200 (29.8)	1691 (41.9)	
CFSS-10 score, mean (SD)	1.17 (1.44)	2.44 (1.54)	0.65 (0.88)	0.67 (1.14)	<.001
CD-RISC score, mean (SD)	59.7 (17.7)	58.7 (18.8)	60.3 (16.9)	59.9 (17.6)	.08
Changes in CD-RISC score, mean (SD)	2.5 (24.6)	5.5 (24.4)	5.2 (23.4)	−1.5 (25.0)	<.001
Age, mean (SD), y	71.0 (6.1)	70.9 (6.2)	69.7 (5.3)	72.0 (6.4)	<.001
Age group, y					
60-64	417 (10.3)	127 (30.5)	163 (39.1)	127 (30.5)	<.001
65-69	1465 (36.3)	410 (28.0)	490 (33.5)	565 (38.6)	
70-74	1149 (28.5)	320 (27.9)	349 (30.4)	480 (41.8)	
75-79	603 (15.0)	177 (29.4)	133 (22.1)	293 (48.6)	
80-84	266 (6.6)	64 (24.1)	48 (18.1)	154 (57.9)	
≥85	133 (3.3)	44 (33.1)	17 (12.8)	72 (54.1)	
Gender					
Men	1776 (44.0)	481 (27.1)	537 (30.2)	758 (42.7)	.30
Women	2257 (56.0)	661 (29.3)	663 (29.4)	933 (41.3)	
Marital status					
Never married	21 (0.5)	6 (28.6)	9 (42.9)	6 (28.6)	.007
Married	3401 (84.3)	936 (27.5)	1049 (30.8)	1416 (41.6)	
Divorced	39 (1.0)	13 (33.3)	9 (23.1)	17 (43.6)	
Widowed	572 (14.2)	187 (32.7)	133 (23.3)	252 (44.1)	
Education					
Never attended school	1642 (40.7)	449 (27.3)	426 (25.9)	767 (46.7)	<.001
Primary school	1437 (35.6)	411 (28.6)	457 (31.8)	569 (39.6)	
Middle school	744 (18.5)	217 (29.2)	249 (33.5)	278 (37.4)	
High school or equivalent	175 (4.3)	60 (34.3)	56 (32.0)	59 (33.7)	
College or above	35 (0.9)	5 (14.3)	12 (34.3)	18 (51.4)	
Monthly income					
<2000 CNY	349 (8.7)	111 (31.8)	77 (22.1)	161 (46.1)	.006
2000-5000 CNY	3505 (86.9)	981 (28.0)	1079 (30.8)	1445 (41.2)	
>5000 CNY	179 (4.4)	50 (27.9)	44 (24.6)	85 (47.5)	
Smoking status					
Nonsmoker	3065 (76.0)	883 (28.8)	872 (28.5)	1310 (42.7)	.02
Smoker	714 (17.7)	186 (26.1)	249 (34.9)	279 (39.1)	
Ex-smoker	254 (6.3)	73 (28.7)	79 (31.1)	102 (40.2)	
Alcohol consumption					
No	3367 (83.5)	963 (28.6)	981 (29.1)	1423 (42.3)	.15
Yes	666 (16.5)	179 (26.9)	219 (32.9)	268 (40.2)	
Physical exercise					
No exercise	1320 (32.7)	431 (32.7)	327 (24.8)	562 (42.6)	<.001
Irregular exercise	927 (23.0)	276 (29.8)	267 (28.8)	384 (41.4)	
Exercise regularly	1786 (44.3)	435 (24.4)	606 (33.9)	745 (41.7)	
SP score, mean (SD)	13.5 (4.6)	13.4 (4.6)	13.9 (4.6)	13.3 (4.5)	.006
No. of chronic diseases					
0	1000 (24.8)	230 (23.0)	365 (36.5)	405 (40.5)	<.001
1	1421 (35.2)	392 (27.6)	415 (29.2)	614 (43.2)	
2	910 (22.6)	260 (28.6)	254 (27.9)	396 (43.5)	
≥3	702 (17.4)	260 (37.0)	166 (23.7)	276 (39.3)	

Abbreviations: CD-RISC, Connor-Davidson Resilience Scale; CFSS-10, Chinese Frailty Screening Scale-10; CNY, Chinese Yuan (1 CNY = US $0.145); SP, social participation.

### Statistical Analysis

Descriptive analyses were conducted to outline participant characteristics. Continuous variables were presented as mean (SD), while categorical variables were expressed as counts and percentages. Changes in PR (ΔPR) were calculated using the difference between follow-up and baseline CD-RISC scores. Frailty progression was classified into 3 categories: improvement, stability, and deterioration. For instance, a follow-up score that is lower than the baseline score indicates improvement. Univariate correlations between frailty progression and baseline characteristics were assessed using 1-way analysis of variance for continuous variables and the χ^2^ test for categorical variables.

Furthermore, the PR score was standardized by subtracting the mean from the original data and then dividing by the SD, and ΔPR score was transformed by dividing by SD. Initially, linear regression was used to examine the longitudinal correlations between PR and ΔPR with frailty, whereas logistic regression was used to estimate the odds ratio (OR) of changes in each SD for both PR and ΔPR as independent variables of frailty progression. Subsequently, nonlinear associations between PR and ΔPR with frailty and frailty progression were analyzed using restricted cubic spline (RCS) analyses with 5 knots at the 5th, 27.5th, 50th, 72.5th, and 95th percentiles.^30^ Moreover, the interaction between PR and ΔPR with baseline frailty was explored. The standardized coefficients (β), ORs, and their 95% CI for each model were presented, adjusting for all covariates and baseline frailty. Statistically, all analyses were performed using Stata 14 (StataCorp) from May to July 2024, with significance set at *P* < .05 for 2-tailed tests.

## Results

### Baseline Characteristics and Frailty Progression

Among the 4033 older adults aged 60 years and above included in the analysis, 2257 (56.0%) were women, 3401 (84.5%) were married, 3079 (76.3%) had completed only primary school education or less; and mean (SD) age was 71.0 (6.1) years. Additionally, 3505 (86.9%) reported a monthly income between 2000 and 5000 Chinese Yuan (CNY; 1 CNY = US $0.145). Notably, 3065 participants (76.0%) were nonsmokers, and 3367 (83.5%) did not consume alcohol. Furthermore, 1786 participants (44.3%) reported exercising regularly, and 1612 (40.0%) had more than 1 chronic illness. In addition, a comparison of baseline characteristics between included and excluded participants indicated that those excluded had higher levels of frailty, but no significant difference in PR. Several covariates exhibited notable differences between the 2 groups (eTable 3 in Supplement 1).

During the 3-year follow-up period, outcomes related to frailty progression indicated that 1142 participants (28.3%) experienced improvement, 1200 (29.8%) remained stable, and 1691 (41.9%) experienced deterioration. With the exception of the baseline CD-RISC score, gender, and alcohol consumption, all other variables showed significant associations with frailty progression (Table 1).

### Associations of PR and ΔPR With Frailty

**Figure 2.**
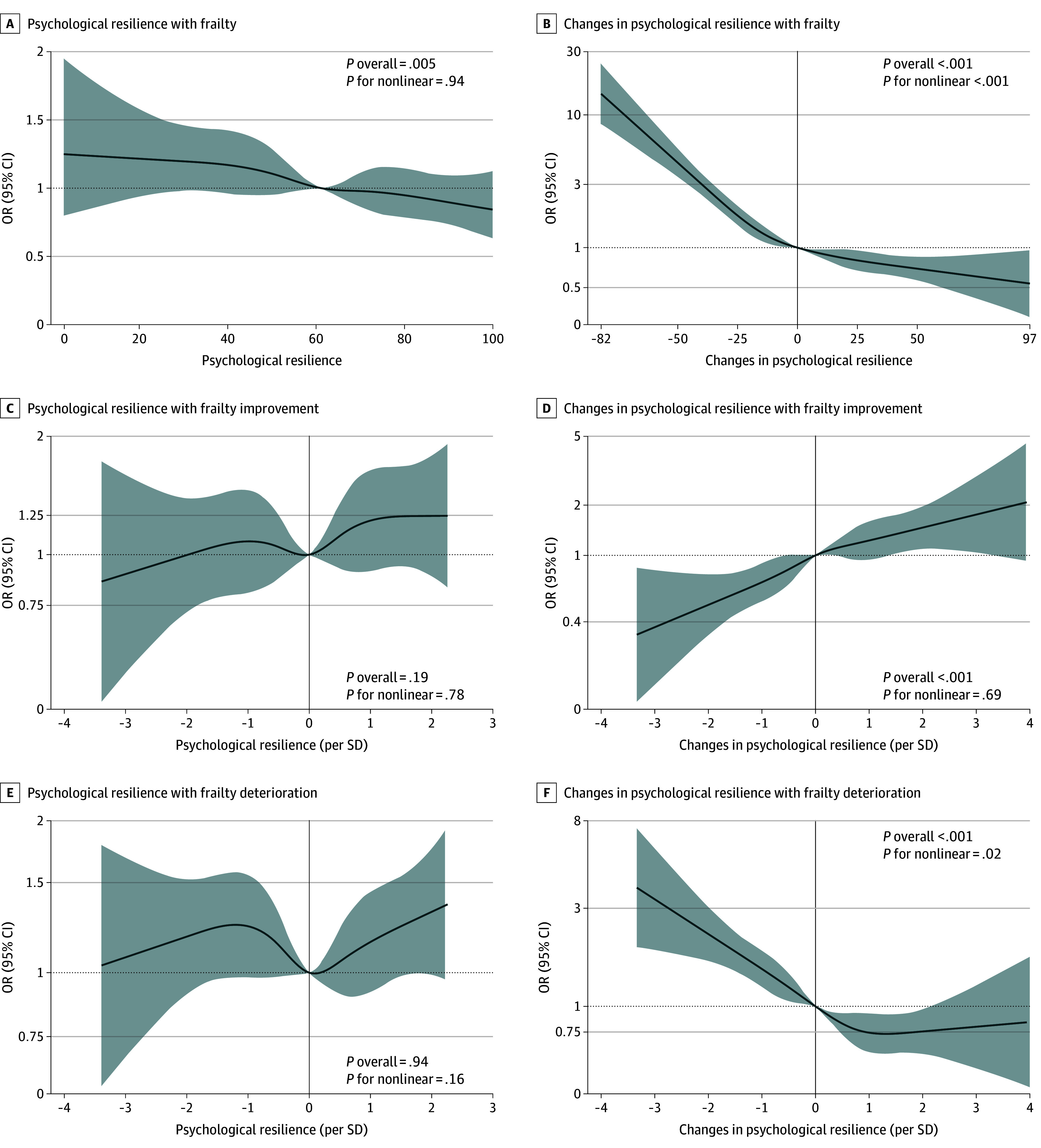
Longitudinal Associations Between Psychological Resilience and Its Changes With Frailty and Frailty Progression Change in psychological resilience was calculated by difference in psychological resilience score between follow-up and baseline. Solid lines indicate the odds ratios (ORs); shaded areas, 95% CIs.

At baseline, the results show a significant association between PR and frailty among the included participants (β, −0.079 [95% CI, −0.108 to −0.049]; *P* < .001), as well as among the excluded participants and in the overall sample (eTable 4 in Supplement 1). Furthermore, the longitudinal associations between PR and ΔPR with frailty are detailed in Table 2. A significant association existed between PR and follow-up frailty, with a notable interaction with baseline frailty. Specifically, PR exhibited a more pronounced association with follow-up frailty in older adults with a baseline frailty score of at least 3 (β, −0.136 [95% CI, −0.214 to −0.057]; *P* = .001) compared with those with a score of 2 or less (β, −0.020 [−0.052 to 0.012]; *P* = .23). There was not a significant nonlinear association between PR and frailty (Figure 2A).

**Table 2.** Longitudinal Associations Between PR and Its Changes With Frailty^a^

Independent variables	Overall			Baseline frailty score ≤2			Baseline frailty score ≥3			*P* for interaction
	β (95% CI)	*P* value	*P* for nonlinear	β (95% CI)	*P* value	*P* for nonlinear	β (95% CI)	*P* value	*P* for nonlinear	
PR	−0.042 (−0.072 to −0.013)	.005	.94	−0.020 (−0.052 to 0.012)	.23	.78	−0.136 (−0.214 to −0.057)	.001	.84	<.001
ΔPR	−0.196 (−0.224 to −0.168)	<.001	<.001	−0.208 (−0.239 to −0.177)	<.001	<.001	−0.187 (−0.260 to −0.115)	<.001	.04	.24
ΔPR										
<0	−0.186 (−0.228 to −0.145)	<.001	NA	−0.176 (−0.223 to −0.130)	<.001	NA	−0.220 (−0.329 to −0.111)	<.001	NA	.11
>0	−0.068 (−0.107 to −0.029)	.001	NA	−0.054 (−0.099 to −0.010)	.02	NA	−0.126 (−0.231 to −0.022)	.02	NA	.03

Abbreviations: NA, not applicable; PR, psychological resilience; ΔPR, changes in psychological resilience.

^a^ Models were adjusted for age, gender, marital status, education, monthly income, smoking status, alcohol consumption, physical exercise, social participation, number of chronic diseases, and baseline frailty.

Similarly, ΔPR showed a significant association with follow-up frailty, although no significant interaction was observed between ΔPR and baseline frailty. The RCS analysis indicated that nonlinear associations between ΔPR and follow-up frailty were significant (Figure 2B) and remained significant among older adults with varying levels of baseline frailty. Specifically, using 0 as the cutoff point, the association between ΔPR and follow-up frailty (β, −0.186 [95% CI, −0.228 to −0.145]; *P* < .001) was found to be stronger when ΔPR was less than 0 compared with the association when ΔPR was greater than 0 (β, −0.068 [95% CI, −0.107 to −0.029]; *P* = .001). Furthermore, the interaction between ΔPR and baseline frailty reached significance only when PR increased, revealing a more substantial association with follow-up frailty among older adults with a baseline frailty score of at least 3 compared with those with a score of 2 or less.

### Associations of PR and ΔPR With Frailty Improvement

The associations between PR and ΔPR with frailty improvement are summarized in Table 3. Logistic regression analyses indicated that PR was positively associated with frailty improvement (OR, 1.28 [95% CI, 1.05-1.57]; *P* = .02), particularly among older adults with a baseline frailty score of at least 3 with a significant interaction. Notably, ΔPR exhibited a positive association with frailty improvement overall (OR, 1.30 [95% CI, 1.19-1.42]; *P* < .001), as well as in both PR decrease and increase. However, no significant nonlinear associations were observed for PR or ΔPR (Figure 2C and 2D). The interaction between ΔPR and frailty improvement was significant only among older adults experiencing PR decrease. Furthermore, the ORs for ΔPR were substantially higher in individuals with a baseline frailty score of at least 3 compared with those with a score of 2 or less.

**Table 3.** Associations Between PR and Its Changes With Frailty Progression^a^

Independent variables	Overall			Baseline frailty score ≤2			Baseline frailty score ≥3			*P* for interaction
	OR (95% CI)	*P* value	*P* for nonlinear	OR (95% CI)	*P* value	*P* for nonlinear	OR (95% CI)	*P* value	*P* for nonlinear	
**Frailty improvement**										
PR	1.06 (0.97-1.16)	.19	.13	0.99 (0.89-1.09)	.79	.30	1.28 (1.05-1.57)	.02	.85	<.001
ΔPR	1.30 (1.19-1.42)	<.001	.69	1.31 (1.18-1.46)	<.001	.37	1.45 (1.18-1.77)	<.001	.77	.13
ΔPR										
<0	1.44 (1.13-1.83)	.003	NA	1.25 (0.93-1.67)	.14	NA	1.80 (1.11-2.94)	.02	NA	.01
>0	1.20 (1.00-1.43)	.04	NA	1.13 (0.91-1.39)	.27	NA	1.88 (1.11-3.18)	.02	NA	.33
**Frailty deterioration**										
PR	1.00 (0.93-1.08)	.94	.16	1.03 (0.95-1.11)	.53	.17	0.87 (0.70-1.08)	.21	.88	.002
△PR	0.74 (0.69-0.80)	<.001	.02	0.76 (0.70-0.81)	<.001	.005	0.60 (0.47-0.75)	<.001	.67	.05
ΔPR										
<0	0.65 (0.55-0.78)	<.001	NA	0.70 (0.57-0.85)	<.001	NA	0.49 (0.29-0.82)	.007	NA	.02
>0	0.88 (0.76-1.03)	.11	NA	0.94 (0.80-1.11)	.46	NA	0.45 (0.23-0.88)	.02	NA	.11

Abbreviations: NA, not applicable; OR, odds ratio; PR, psychological resilience; ΔPR, changes in psychological resilience.

^a^ Models were adjusted for age, gender, marital status, education, monthly income, smoking status, alcohol consumption, physical exercise, social participation, number of chronic diseases, and baseline frailty.

### Associations of PR and ΔPR With Frailty Deterioration

Table 3 displays the associations of PR and ΔPR with frailty deterioration. The analyses indicated that PR did not have significant linear or nonlinear associations with frailty deterioration (Figure 2E). Nevertheless, a significant interaction was identified, showing lower ORs among older adults with a baseline frailty score of at least 3 compared with those with a score of 2 or less. In contrast, ΔPR had a negative association with frailty deterioration (OR, 0.74 [95% CI, 0.69-0.80]; *P* < .001) and showed significant nonlinear association (Figure 2F). Although the interaction between ΔPR and baseline frailty was significant only among older adults experiencing PR decrease, the ORs for ΔPR were lower in those with a baseline frailty score of at least 3 compared with those with a score of 2 or less.

## Discussion

The findings of this study indicated that older adults with greater PR exhibited both cross-sectional and longitudinal associations with lower levels of frailty. These results were consistent with previous cross-sectional studies conducted in a community setting,^19^ hospital setting,^20^ and nursing home.^22^ To our knowledge, the present study is the first to find a longitudinal association between PR and frailty in a community setting. Generally, exposure to adversity is a robust risk factor of disruptions in psychological functioning.^31^ Furthermore, resilience encompasses not only psychological well-being but also physical functioning. Prior longitudinal studies similarly indicated that greater PR was associated with improved walkability and gait speed,^12,14^ key indicators of physical frailty.^32^ Our findings underscored the notion that older adults with greater PR were at a lower risk of frailty, reinforcing the characteristic of resistance to adversity associated with PR and affirming its role as an important health factor.^33^

Furthermore, our study found significant associations between PR and its changes with future frailty and frailty progression. It highlighted nonlinear associations among these variables and emphasized the differential effects observed in older adults with varying baseline frailty levels. First, we found a linear association between baseline PR and future frailty, while ΔPR exhibited a nonlinear association with future frailty. Interestingly, the association of ΔPR with future frailty was more pronounced among older adults experiencing PR decline compared with those showing improvement. Similarly, ΔPR was significantly related to both improvement and deterioration in frailty, with a more substantial association observed in cases of PR decline. To our knowledge, only 1 longitudinal study has previously explored this association, observing differences in CD-RISC scores of 0.56 and −0.64 between the remained-robust group and the worsened-frailty group among older adults; however, the result was not statistically significant.^24^ This lack of significance may be attributed to a short observational period and a small simple size.^24^ Although frailty is generally understood as a dynamic process, more pronounced changes were likely to manifest over a longer timeframe.^34,35,36^ In addition, fluctuations in PR may be challenging to capture within a 6-month follow-up in the absence of major life events.^24^ Despite being considered a relatively stable trait, PR is part of a dynamic, adaptive process that carries crucial implications for the promotion and maintenance of health and well-being in later life.^15^ Experts assert that individuals can enhance their resilience, thereby contributing to improved health outcomes and facilitate recovery during illness.^33^ Previous research indicated that interventions designed to bolster PR had the potential to enhance overall health and well-being.^37^ Notably, PR decline among older adults may signify a trend toward increased risk. These findings emphasized the importance of monitoring PR changes as a strategy for forecasting future frailty progression, particularly for those experiencing declines in PR.

Second, we found that PR and baseline frailty levels interacted in their associations with future frailty, whereas the interaction with ΔPR was not significant. This interaction persisted in the associations between PR and both frailty improvement and deterioration, suggesting that PR was more strongly associated with future frailty and frailty progression among older adults with initially higher levels of frailty. These results reinforce the fundamental attribute of PR—the ability to bounce back—and align with a prior national study involving 10 753 US residents aged 51 to 98 years, which found that higher levels of resilience buffered the negative impacts of new chronic conditions on physical functioning limitations later in life.^38^ Additionally, another observational study derived from a multisite RCT demonstrated that increases in mean gait speed and walking distance were greater in the highly resilient group following a minimal trauma hip fracture than in the least resilient group.^14^ These findings supported a resilience framework that emphasizes intraindividual resources as potential interventions between negative adversity and subsequent well-being.^11,30,37^ Importantly, our results also suggested that older adults with frailty symptoms exhibited heightened sensitivity to variations in resilience, implying that frail older individuals with lower PR were at a higher risk of frailty deterioration. This increased risk may stem from individuals with frailty being more at risk for external stressors,^32^ which can amplify the effects of PR in the absence of resources that mitigate these stressors and promote recovery.^11^ Resilient older adults with frailty are better equipped to return to their baseline state, while those with lower resilience face a greater risk of adverse outcomes. Although not all interactions were statistically significant, the results across segment groups maintained a consistent direction, suggesting that ΔPR may have a more pronounced association with future frailty and frailty progression among older adults with high baseline frailty compared with those with lower baseline frailty.

In addition to the main findings, our study also reported on the prevalence of PR, with a mean CD-RISC score of 59.7 among a large sample of community-dwelling older people. Previous surveys conducted in Singapore revealed a high level of resilience, with a mean CD-RISC score of 84.9 among community-dwelling older Chinese adults,^39^ whereas a community sample of older adults in Korea showed a lower level of resilience, with a mean CD-RISC score of 50.3.^40^ PR among older populations may tend to be stronger in regions with higher socioeconomic status, which can provide more accessible resources that offer valued supports and services.^41^ In the context of rapid population aging, the role of resilience in healthy aging has garnered increasing attention^6^; however, there are few comparable studies, which limits the understanding of resilience within the framework of healthy aging. There is a need for more standardized research, particularly across different countries and ethnic groups.

### Strengths and Limitations

This study contributed to the limited body of prospective research examining the associations between PR and frailty progression using a large population-based cohort. This focus aligned with the growing recognition that resilience and frailty are dynamic and can vary over time.^15,34^ Moreover, we found nonlinear associations between ΔPR and frailty as well as frailty deterioration. Subgroup and segment analyses enhanced the robustness of our results, providing a more comprehensive understanding of the association between PR and frailty. We also controlled for multiple important confounders, including age, education, health behaviors, and social participation, thereby increasing the reliability of our results.

However, it is important to acknowledge limitations of this study. First, the choice of frailty assessment tool may influence the results. The CFSS-10, developed and validated specifically within the Chinese context, may constrain the global applicability of the results. Moreover, as the CFSS-10 incorporates both physical and mental aspects of frailty, it may inadvertently strengthen the observed association between PR and frailty. While the concept of frailty is evolving toward a multidimensional approach,^42^ utilizing the Fried frailty phenotype^32^ may provide a more effective method for examining the association between PR and physical frailty. Second, although our sample size was relatively large, all participants were drawn from the same region, which necessitates caution in generalizing the findings. Further research is needed in diverse socioeconomic contexts and among varied ethnic populations. Third, selection bias may pose a concern in our analyses due to the exclusion of 539 participants (11.8%), although this remains within an acceptable range. However, considering that the association between PR and frailty has been well established in older patients with poorer health,^16,20,22^ coupled with the presence of competitive mortality, it is plausible that the observed association may be underestimated. Fourth, as the study was initiated in the midst of the COVID-19 pandemic, the baseline characteristics of the participants may have been affected. While previous studies have indicated that older adults exhibited greater stability in mental health and enhanced emotional resilience during the pandemic compared to younger adults,^43,44,45^ lifestyle factors such as social participation and physical exercise may have declined from their usual levels, potentially influencing the adjusted results. Additionally, we evaluated the association between PR and frailty while controlling for multiple covariates; however, it remained possible that these variables may mediate the observed associations. Therefore, we recommend that future studies explore the potential mediating effects.

## Conclusions

The findings contribute to the limited evidence regarding the association between PR and frailty among community-dwelling older adults. The study found that greater PR was associated with a lower risk of frailty, particularly among frail older adults. Notably, individuals experiencing declines in PR exhibited more pronounced associations on future frailty and frailty progression compared with those with increasing PR, indicating a nonlinear association. This study offers valuable insights into the association between PR and frailty in older adults. Monitoring PR changes may help forecast future frailty trajectories, highlighting the need to support individuals facing declines in resilience. Targeted interventions that prioritize enhancing PR hold potential for preventing and ameliorating frailty.

## Notes

Supplement 1. **eTable 1.** The 10-Item Chinese Frailty Screening Scale (CFSS-10) **eTable 2.** Content of the Connor-Davidson Resilience Scale (CD-RISC) **eTable 3.** Comparison of Characteristics of Included and Excluded Samples **eTable 4.** Cross-Sectional Associations Between Psychological Resilience and Frailty in Included and Excluded Samples at Baseline

Supplement 2.
Data Sharing Statement
