# Supplementary material for: Psychological Resilience and Frailty Progression in Older Adults
Source: JAMA Netw Open. 2024 Nov 25;7(11):e2447605. doi: 10.1001/jamanetworkopen.2024.47605 (PMC11589792; doi:10.1001/jamanetworkopen.2024.47605)
Supplement: Supplement 1. — eTable 1. The 10-Item Chinese Frailty Screening Scale (CFSS-10) eTable 2. Content of the Connor-Davidson Resilience Scale (CD-RISC) eTable 3. Comparison of Characteristics of Included and Excluded Samples eTable 4. Cross-Sectional Associations Between Psychological Resilience and Frailty in Included and Excluded Samples at Baseline [file jamanetwopen-e2447605-s001.pdf]

Supplementary Online Content

Ye B, Li Y, Bao Z, Gao J. Psychological resilience and frailty progression in older adults. *JAMA Netw Open*. 2024;7(11):e2447605. doi:10.1001/jamanetworkopen.2024.47605

eTable 1. The 10-Item Chinese Frailty Screening Scale (CFSS-10)

eTable 2. Content of the Connor-Davidson Resilience Scale (CD-RISC)

eTable 3. Comparison of Characteristics of Included and Excluded Samples

eTable 4. Cross-Sectional Associations Between Psychological Resilience and Frailty in Included and Excluded Samples at Baseline

© 2024 Ye B et al. *JAMA Network Open*.

eTable 1. The Chinese frailty screening scale-10 (CFSS-10)

Item	Question	Answer
Illnesses	Have you been diagnosed with at least 5 illnesses by doctors? (i.e., Hypertension; Dyslipidemia; Diabetes or high blood sugar; Cancer or malignant tumor (excluding minor skin cancers); Chronic lung diseases; Liver disease; Heart attack, coronary heart disease, angina, congestive heart failure, or other heart problems; Stroke; Kidney disease; Stomach or other digestive disease; Alzheimer's or Parkinson's disease; Arthritis or rheumatism; Asthma)	□ Yes<br>□ No
Exhaustion	Did you often feel tired or fatigue in the last month?	□ Yes<br>□ No
Lack of appetite	In the last three months, did you eat less due to loss of appetite, indigestion, teeth problem or dysphagia?	□ Yes<br>□ No
Visual impairment	Do you experience problems in your daily life due to poor vision?	□ Yes<br>□ No
Hearing loss	Do you experience problems in your daily life due to poor hearing?	□ Yes<br>□ No
Resistance	Do you have difficulty with climbing 10 stairs or a flight without resting?	□ Yes<br>□ No
Physical inactivity	Did you walk for at least 10 minutes or 400 meters continuously in the last week?	□ Yes<br>□ No
Attention	Did you often wander or have difficulty with concentrating in the last month?	□ Yes<br>□ No
Orientation	Did you frequently get the date wrong or get lost in the last month?	□ Yes<br>□ No
Depressive symptom	Did you feel you were not interested in doing anything in the last month?	□ Yes<br>□ No

© 2024 Ye B et al. *JAMA Network Open*.

eTable 2. Content of the Connor-Davidson resilience scale (CD-RISC)

Item no.	Description
1	Able to adapt to change
2	Close and secure relationships
3	Sometimes fate or God can help
4	Can deal with whatever comes
5	Past success gives confidence for new challenge
6	See the humorous side of things
7	Coping with stress strengthens
8	Tend to bounce back after illness or hardship
9	Things happen for a reason
10	Best effort no matter what
11	You can achieve your goals
12	When things look hopeless, I don't give up
13	Know where to turn for help
14	Under pressure, focus and think clearly
15	Prefer to take the lead in problem solving
16	Not easily discouraged by failure
17	Think of self as strong person
18	Make unpopular or difficult decisions
19	Can handle unpleasant feelings
20	Have to act on a hunch
21	Strong sense of purpose
22	In control of your life
23	I like challenges
24	You work to attain your goals
25	Pride in your achievements

Note: All of which carry a 5-point range of responses, as follows: ***not true at all (0), rarely true (1), sometimes true (2), often true (3), and true nearly all of the time (4)***. The scale is rated based on how the subject has felt over the past month. The total score ranges from 0~100, with higher scores reflecting greater resilience.

© 2024 Ye B et al. *JAMA Network Open.*

eTable 3. Comparison of characteristics of included and excluded samples

Independent variables		Included sample (N=4033)	Excluded sample (N=539)	P value
CFSS-10 score, mean (SD)		1.17 (1.44)	1.65 (1.83)	<0.001
CD-RISC score, mean (SD)		59.68 (17.75)	58.33 (19.17)	0.10
Age, years, mean (SD)		71.02 (6.11)	74.38 (8.10)	<0.001
Age group, years	60-64	417 (10.3)	36 (6.7)	<0.001
	65-69	1465 (36.3)	146 (27.1)	
	70-74	1149 (28.5)	118 (21.9)	
	75-79	603 (15.0)	109 (20.3)	
	80-84	266 (6.6)	56 (10.4)	
	85+	133 (3.3)	73 (13.6)	
Gender	Men	1776 (44.0)	273 (50.6)	0.004
	Women	2257 (56.0)	266 (49.4)	
Marital status	Never married	21 (0.5)	6 (1.1)	<0.001
	Married	3401 (84.3)	400 (74.2)	
	Divorced	39 (1.0)	18 (3.3)	
	Widowed	572 (14.2)	115 (21.3)	
Education	Never went to school	1642 (40.7)	258 (47.9)	<0.001
	Primary school	1437 (35.6)	149 (27.6)	
	Middle school	744 (18.5)	91 (16.9)	
	High school or equivalent	175 (4.3)	28 (5.2)	
	College or above	35 (0.9)	13 (2.4)	
Monthly income	<2000 CNY	349 (8.7)	67 (12.4)	0.005
	2000-5000 CNY	3505 (86.9)	441 (81.8)	
	>5000 CNY	179 (4.4)	31 (5.8)	
Smoking	Non-smoker	3065 (76.0)	411 (76.3)	0.21
	Smoker	714 (17.7)	85 (15.8)	
	Ex-smoker	254 (6.3)	43 (8.0)	
Drinking	No	3367 (83.5)	456 (84.6)	0.51
	Yes	666 (16.5)	83 (15.4)	
Physical exercise	No exercise	1320 (32.7)	203 (37.7)	0.006
	Irregular exercise	927 (23.0)	136 (25.2)	
	Exercise regularly	1786 (44.3)	200 (37.1)	
SP score, mean (SD)		13.52 (4.55)	13.28 (5.17)	0.28
Number of chronic diseases	0	1000 (24.8)	116 (21.5)	0.003
	1	1421 (35.2)	166 (30.8)	
	2	910 (22.6)	134 (24.9)	
	3+	702 (17.4)	123 (22.8)	

CFSS-10: Chinese Frailty Screening Scale-10; CD-RISC: SD: Standard Deviation; Connor-Davidson Resilience Scale; CNY: China Yuan (1 CNY = 0.145 US dollars); SP: Social Participant.

© 2024 Ye B et al. *JAMA Network Open.*

eTable 4. Cross-sectional associations between psychological resilience and frailty in included and excluded samples at baseline.

Independent variables		Overall (n=4572)		Included sample (n=4033)		Excluded sample (n=539)	
		β (95%CI)	P value	β (95%CI)	P value	β (95%CI)	P value
CD-RISC score		-0.090 (-0.118, -0.062)	<0.001	-0.079 (-0.108, -0.049)	<0.001	-0.168 (-0.256, -0.080)	<0.001
Age, year		0.201 (0.170, 0.231)	<0.001	0.203 (0.172, 0.235)	<0.001	0.107 (0.012, 0.202)	0.03
Women		0.072 (0.036, 0.108)	<0.001	0.067 (0.028, 0.106)	0.001	0.131 (0.033, 0.228)	0.009
Marital status	Never married	0.010 (-0.017, 0.036)	0.47	0.008 (-0.021, 0.036)	0.59	0.029 (-0.048, 0.106)	0.46
	Married	1 (ref)		1 (ref)		1 (ref)	
	Divorced	0.033 (0.007, 0.060)	0.02	0.037 (0.008, 0.065)	0.01	0.005 (-0.071, 0.081)	0.90
	Widowed	0.028 (0.000, 0.057)	0.05	0.020 (-0.010, 0.051)	0.19	0.062 (-0.026, 0.151)	0.17
Education	Never went to school	1 (ref)		1 (ref)		1 (ref)	
	Primary school	0.012 (-0.020, 0.043)	0.47	0.020 (-0.013, 0.054)	0.23	-0.044 (-0.134, 0.045)	0.33
	Middle school	0.010 (-0.022, 0.042)	0.55	0.016 (-0.018, 0.050)	0.36	-0.020 (-0.110, 0.071)	0.67
	High school or equivalent	0.037 (0.008, 0.067)	0.01	0.040 (0.008, 0.071)	0.01	0.007 (-0.081, 0.095)	0.88
	College or above	-0.008 (-0.036, 0.02)	0.58	-0.016 (-0.046, 0.014)	0.30	-0.001 (-0.096, 0.093)	0.97
Monthly income	<2000 CNY	1 (ref)		1 (ref)		1 (ref)	
	2000-5000 CNY	-0.039 (-0.072, -0.007)	0.02	-0.056 (-0.091, -0.021)	0.002	0.058 (-0.035, 0.152)	0.22
	>5000 CNY	-0.036 (-0.070, -0.002)	0.04	-0.055 (-0.092, -0.019)	0.003	0.074 (-0.030, 0.178)	0.16
Smoking	Non-smoker	1 (ref)		1 (ref)		1 (ref)	
	Smoker	-0.014 (-0.047, 0.020)	0.42	-0.015 (-0.051, 0.021)	0.43	-0.014 (-0.108, 0.080)	0.77
	Ex-smoker	0.007 (-0.022, 0.036)	0.64	0.001 (-0.031, 0.033)	0.97	0.042 (-0.042, 0.127)	0.33
Drinking		0.012 (-0.019, 0.043)	0.44	0.010 (-0.023, 0.044)	0.55	0.036 (-0.055, 0.128)	0.43
Physical exercise	No exercise	1 (ref)		1 (ref)		1 (ref)	
	Irregular exercise	-0.046 (-0.077, -0.016)	0.003	-0.050 (-0.083, -0.018)	0.003	-0.027 (-0.115, 0.061)	0.55
	Exercise regularly	-0.129 (-0.160, -0.097)	<0.001	-0.129 (-0.163, -0.096)	<0.001	-0.111 (-0.206, -0.016)	0.02
SP score		-0.049 (-0.077, -0.022)	<0.001	-0.042 (-0.071, -0.012)	0.006	-0.086 (-0.171, -0.001)	0.05
Number of chronic diseases	0	1 (ref)		1 (ref)		1 (ref)	
	1	0.067 (0.034, 0.101)	<0.001	0.065 (0.029, 0.100)	<0.001	0.087 (-0.014, 0.188)	0.09
	2	0.096 (0.064, 0.129)	<0.001	0.090 (0.055, 0.124)	<0.001	0.141 (0.042, 0.240)	0.005
	3+	0.264 (0.232, 0.296)	<0.001	0.263 (0.229, 0.297)	<0.001	0.262 (0.163, 0.362)	<0.001

© 2024 Ye B et al. *JAMA Network Open*.

CD-RISC: Connor-Davidson Resilience Scale; CNY: China Yuan (1 CNY = 0.145 US dollars); SP: Social Participant; CI: Confidence interval.

© 2024 Ye B et al. *JAMA Network Open*.
