# Supplementary material for: Psychological Resilience and Frailty Progression in Older Adults
Source: JAMA Netw Open. 2024 Nov 25;7(11):e2447605. doi: 10.1001/jamanetworkopen.2024.47605 (PMC11589792; doi:10.1001/jamanetworkopen.2024.47605)
Supplement: Supplement 2. — Data Sharing Statement [file jamanetwopen-e2447605-s002.pdf]

Data Sharing Statement

Ye. Psychological Resilience and Frailty Progression in Older Adults. *JAMA Netw Open*. Published November 25, 2024. doi:10.1001/jamanetworkopen.2024.47605

Data
Data available: Yes
Data types: Deidentified participant data
How to access data: The data applied and analyzed in the current study are available from the corresponding author upon reasonable request.
When available: With publication

Supporting Documents
Document types: None

Additional Information
Who can access the data: Researchers who request data for further scientific research.
Types of analyses: For any purpose.
Mechanisms of data availability: After approval of a proposal.
